# Protocol of an implementation study of a clinician intervention to reduce fear of recurrence in cancer survivors (CIFeR_2 implementation study)

**DOI:** 10.1186/s12909-023-04279-0

**Published:** 2023-05-05

**Authors:** Jia Liu, Anastasia Serafimovska, Sharon He, Phyllis Butow, Joanne Shaw, Christopher John McHardy, Georgia Harris, Zoe Butt, Jane Beith

**Affiliations:** 1 Kinghorn Cancer Centre, St Vincent’s Hospital, Sydney, NSW Australia; 2 School of Psychology, Psycho-Oncology Cooperative Research Group, The University of Sydney, Sydney, NSW Australia; 3 St Vincent’s Clinical School, University of New South Wales, Darlinghurst, NSW Australia; 4 The Chris O’Brien Lifehouse, Camperdown, NSW Australia; 5 Western Sydney University, Campbelltown, NSW Australia

**Keywords:** Cancer, Breast Cancer, Fear of Cancer recurrence, Psycho-Oncology, Communication, Clinician intervention, Training

## Abstract

**Background:**

Fear of cancer recurrence (FCR) affects 50–70% of cancer survivors with 30% reporting an unmet need for help with managing FCR. Patients indicate desire to discuss FCR with clinicians, however clinicians indicate discomfort with managing FCR and no formal educational interventions on how to discuss FCR or worry exists for oncology clinicians. Our team developed a novel clinician-driven brief education intervention to help patients manage FCR (the Clinician Intervention to Reduce Fear of Recurrence (CIFeR) intervention). In earlier work, we demonstrated the feasibility, acceptability, and efficacy of CIFeR in reducing FCR in breast cancer patients. We now aim to explore the barriers and facilitators to implementing this low-cost brief intervention within routine oncology practice in Australia. The primary objective is to assess the adoption of CIFeR in routine clinical practice. Secondary objectives are to identify the uptake and sustainability, perceived acceptability, feasibility, costs, barriers and facilitators of implementation of CIFeR in routine clinical practice, and to assess whether training in CIFeR increases clinicians’ self-efficacy in managing FCR with their patients.

**Methods:**

This multicentre, single-arm Phase I/II implementation study will recruit medical and radiation oncologists and oncology surgeons who treat women with early breast cancer. Participants will complete online CIFeR training. They will then be asked to use CIFeR with suitable patients for the next 6 months. Participants will complete questionnaires prior to, immediately after and 3 and 6 months after training to assess confidence addressing FCR, and 3 and 6 months after training to assess Proctor Implementation outcomes. At 6 months, they will also be asked to participate in a semi-structured telephone interview to elicit their feedback about barriers and facilitators to using CIFeR in routine clinical practice.

**Discussion:**

This study will provide further data to support the routine use of an evidence-based, clinician-lead educational intervention to reduce FCR in breast cancer patients. Additionally, this study will identify any barriers and facilitators to implementing the CIFeR intervention in routine care and evidence for integration of FCR training into oncology communication skills education.

**Trial registration:**

Prospectively registered with the Australian New Zealand Clinical Trials Registry, ACTRN12621001697875.

**Trial sponsor:**

Chris O’Brien Lifehouse.

**Protocol version:**

2.6, Dated 28th February 2023.

**Supplementary Information:**

The online version contains supplementary material available at 10.1186/s12909-023-04279-0.

## Background

Improvements in cancer screening, diagnosis and management has resulted in substantial increased survival rates. Long-term survival is common after treatment for early breast cancer, with 5-year survival rates reaching 89% [1]. One of the most prevalent unmet needs of breast cancer survivors is fear of cancer recurrence (FCR) [2]. FCR is defined as ‘fear associated with the possibility the cancer will return or progress in the same or different part of the body’ [3]. FCR is a significant problem affecting 50–70% of cancer survivors across all cancer subtypes which persists over time [4, 5]. A systematic review of FCR in adult cancer survivors found inconsistent relationships between cancer stage or objective markers of recurrence risk, and patients’ perceived level of FCR. However, there is moderate evidence that cancer survivors who reported higher FCR expressed lower healthcare satisfaction [4]. High levels of FCR also affects patients’ quality of life and productivity and increases resource expenditure and health system utilization [6]. Importantly, 30% of patients surveyed report significant unmet need for help with managing FCR [7].

A number of studies evaluating evidence-based psychologist-delivered interventions, including a randomized trial conducted by this research team of ConquerFear, [8] have demonstrated sustained efficacy in reducing FCR in patients with high baseline fear levels. However, these programs are resource intensive and time-consuming, not acceptable to all patients, and not tailored for the vast majority of patients with mild-moderate FCR, whose fears may be more appropriately managed by clinicians (such as oncologists) within the context of routine consultations. Furthermore, brief, targeted oncologist-delivered FCR interventions have the potential to improve patient-clinician communication and rapport and prevent the development of severe FCR.

Cross-sectional surveys of cancer survivors indicate that many patients report a desire but reluctance to raise FCR with their doctors for fear of appearing ‘ungrateful’ or damaging the patient-physician relationship by suggesting their treatment may not have been successful [9]. Moreover, > 70% of surveyed doctors indicated discomfort with managing FCR [10] with the majority indicating interest in specific methods and education and training on how to better discuss and manage FCR as part of their routine clinical consultation [11]. Current clinical practice guidelines on the identification and management of FCR by Cancer Australia recommends psychological interventions and involvement of family/carers to help address FCR, but provides no recommendations or guidance on how doctors can address FCR with the patient [12]. A systematic review of doctor and nurse-led interventions for managing FCR revealed that no intervention trials currently exist to address FCR in the context of routine consultations [13].

To address these gaps in evidence-based interventions addressing FCR, the investigators developed the 5-component CIFeR intervention based on current knowledge of existing interventions, results of cross-sectional surveys on FCR, FCR theoretical models and expert input (including psycho-oncologists, clinicians, and consumers). The 5-component CIFeR intervention entails: (1) FCR normalisation and reassurance delivered by the clinician during the consultation (2) Provision of concrete prognostic information (if desired by the patient) (3) A take-home education sheet on red-flag recurrence symptoms (4) Brief advice on strategies to manage worry (5) Referral to psychologist if FCR is severe or the patient requests additional help. CIFeR is delivered at any appropriate follow-up appointment to breast cancer survivors who are 6 months to 5 years after completion of treatment (with exception of hormone therapy) for early-stage breast cancer. CIFeR may be delivered either face-to-face, or via TeleHealth.

To determine the usefulness, feasibility and efficacy of CIFeR, we conducted a multicentre, single-arm Phase I/II study involving five oncologists and 61 women with early-stage breast cancer [14]. Patients were surveyed before (T0), one week (T1) and three months (T2) after the intervention on FCR, need for help with FCR and depression/anxiety, and at one week on satisfaction. Oncologists underwent one-hour face-to-face training on the steps and delivery of CIFeR. Overall, 58 women (95%) found CIFeR to be helpful and 59 (98%) would recommend it to others. Women noted that they very much appreciated FCR being addressed by their oncologist and found all components of CIFeR beneficial with 56/58 women (97%) reporting the intervention to be useful and 57/58 (98%) reporting that they would recommend it to other patients. FCR severity, and proportion of women with clinically significant FCR decreased significantly over time. Mean intervention length was 9 min (3–20 min). Average intervention fidelity by the oncologist was 82% (range 67–89%) using audio-recordings of consultations. The intervention was perceived as useful and feasible by oncologists, all of whom have used components of the intervention to help manage FCR in other breast cancer patients. Thus, it was concluded that CIFeR was feasible, acceptable and potentially efficacious.

This brief and low-cost intervention may be effective in preventing FCR, as well as reducing its severity and duration in patients who develop FCR. However, it remains to be demonstrated that clinicians more widely will take up CIFeR in routine clinical practice. To guide implementation efforts, we need to understand the barriers and facilitators of implementing CIFeR in routine care.

Thus, the Clinician Intervention to Reduce Fear of Recurrence (CIFeR_2) study aims to determine the uptake, adoption and sustainability, and perceived acceptability, feasibility, costs, barriers and facilitators to implementation of CIFeR with early-stage breast cancer patients who are 6-months to 5 years after completion of surgery/chemotherapy/radiotherapy.

The resulting data will guide further intervention development, and future large-scale efficacy studies. This sequence of research is recommended by Proctor et al. [15] who position implementation outcomes as preceding both service outcomes and client outcomes, with the latter outcomes being impacted by implementation outcomes. The PARiHS framework (Promoting Action on Research Implementation in Health Services) [16] incorporating strong scientific evidence with a supportive context and implementation facilitation will be used to guide the study.

The primary hypothesis of CIFeR_2 is that:

1. > 50% of participating Medical and Radiation oncologists or surgeons will offer CIFeR to at least 1 early stage/curable breast cancer patient by 3 months after receiving training.

Secondary hypotheses are that:

2. > 50% of participating Medical and Radiation oncologists or surgeons will offer CIFeR to at least 1 early stage/curable breast cancer patient in the last 3 months when surveyed 6-months after their training on the CIFeR intervention.
3. More than 20% of Medical and Radiation oncologists or surgeons invited to join the CIFeR implementation study will agree to participate.
4. Participating Medical and Radiation oncologists or surgeons will deliver 4/5 of the components of CIFeR (80% fidelity) to at least two of the first three patients to whom they deliver CIFeR.
5. Participating Medical and Radiation oncologists or surgeons will find CIFeR acceptable, appropriate and feasible in routine practice.
6. CIFeR implementation will be low in costs across consultation time, resources, and psychologist referrals.
7. Participating Medical and Radiation oncologists or surgeons will report a range of barriers and facilitators to CIFeR implementation in qualitative interviews at 6 months follow-up.
8. There will be no differences between Medical and Radiation oncologists and surgeons with respect to CIFeR implementation outcomes.
9. Oncologists’ or surgeons’ scores on a scale assessing self-efficacy to manage FCR in patients will increase from baseline to post-training, and to 3 and 6-months post-training follow-up.

## Methods/design

This multi-site implementation study is being led by the Chris O’Brien Lifehouse, Sydney Australia in collaboration with the Psycho-Oncology Co-operative Research Group based at the University of Sydney, Australia. This project was prospectively registered with the Australian New Zealand Clinical Trials Registry (ACTRN12621001697875). Ethics approval has been obtained from the St Vincents Hospital Research Ethics Committee (2021/ETH10908).

### Participants

Participants will be Medical and Radiation oncologists and Surgeons, including oncology and surgical senior trainees who treat women with early-stage breast cancer. Oncologists or surgeons will be eligible if they are:

1. Currently practising medical and radiation oncologists and breast surgeons or senior trainees and breast surgical trainees (with > 6 months training in clinical oncology at the time of recruitment) who treat women with early-stage breast cancer.
2. Ability to commit to the study requirements and undertake online CIFeR training modules.

Participants will be recruited through advertisements posted by breast cancer organisations (e.g. the Medical Oncology Group of Australia and Breast Cancer Trials Group) via email and through newsletters as per those organisations’ procedures; by email from the researchers; and by snow-balling recruitment techniques (participants informing colleagues about the study) and social media (e.g. professional Twitter accounts). Where possible, we will obtain estimates of the number of oncologists approached. Interested oncologists will be provided with the research assistant’s email if they would like to speak to a study staff member to obtain further information, and a link to the online Qualtrics portal where they will be provided with an information sheet and provide written online consent.

### Procedure

Participants will be prompted to complete the online (Qualtrics) baseline questionnaire eliciting demographic and practice details, estimated proportion of patients referred to psychologists or other psychosocial health professionals for help with FCR over the past 3 months, and self-efficacy to manage FCR in patients (Supplementary File 1). Oncologists or surgeons who have not responded to invitations to participate or do not complete the baseline assessment will receive up to two emails and two phone calls from the research assistant to prompt completion spaced out over two weeks.

After completing the baseline measure, participants will then be emailed a link to the online CIFeR training, which will be indefinitely available to oncologists or surgeons, allowing them to refresh their familiarity with CiFeR content at their convenience. Upon training completion, participants will click on a link redirecting them to the Qualtrics portal, where they will be asked to complete the post-training self-efficacy measure for managing FCR (Supplementary File 2). Participants will then be asked to use CIFeR with suitable patients for the next six months. An example script for participants to use in their consultations with patients when introducing CIFeR will be provided to participants. They will also be provided with patient hand-outs, online links to CIFeR resources and a 5-point checklist (paper or online version) (Supplementary File 3) which they will be asked to complete after delivering CIFeR to three patients, to assess intervention fidelity.

Given CIFeR will be offered to patients as part of routine care (with no patient-reported outcomes), patients will not need to be consented to the study. Oncologists or surgeons will identify patients suitable for the CIFeR intervention by asking each patient as they attend for follow-up “Do you ever worry that your cancer may come back?” All patients who indicate any worry can be offered CIFeR. If the clinician determines during the consultation that the patient is significantly distressed by FCR, they will refer the patient to a psychosocial health professional or other intervention as per usual practice.

**Fig. 1:**
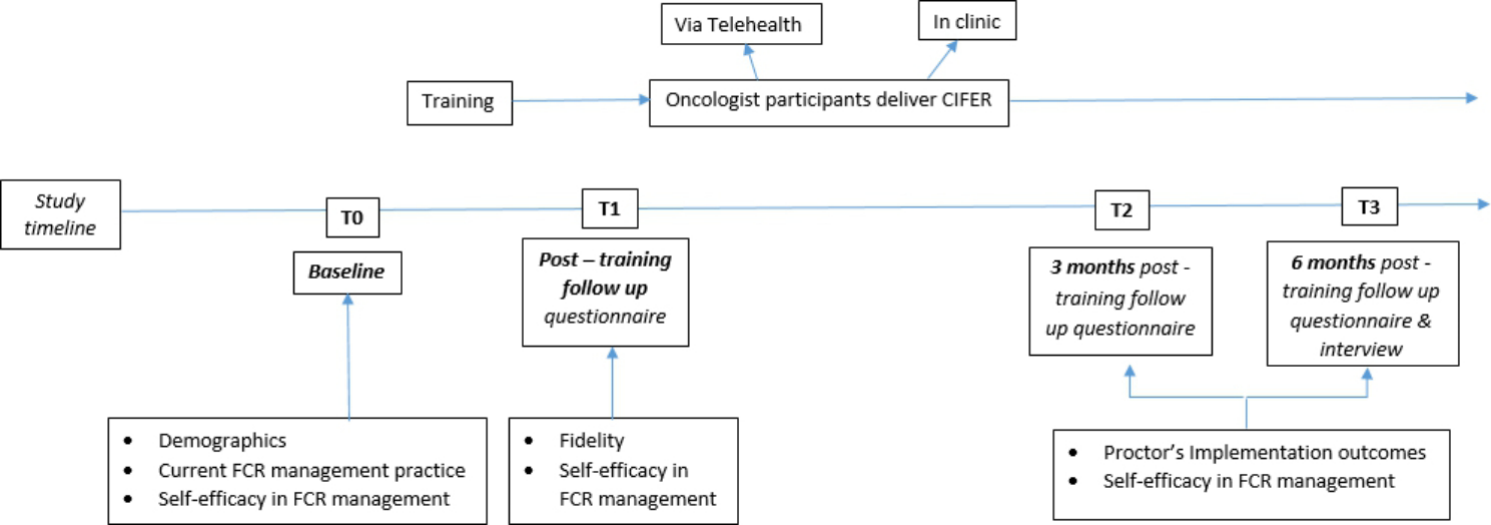
Summary of the study schema and data collected at different timepoints

A research assistant will contact participants 1 month after training completion by phone to prompt them to utilise CIFeR with their patients in follow-up, and to complete the checklist after 3 patients have received CIFeR. At 3 and 6-months post training participants will be emailed a link to an online questionnaire assessing Proctor outcomes, number of patients with whom CIFeR has been used and their self-efficacy in FCR management (Supplementary File 4). Participants who have not completed follow-up measures within 10 days will receive two emails and two phone calls to prompt completion spaced out over two weeks. See Fig. 1 for study schema.

### Training

The CIFeR training has been developed by an expert panel of FCR experts, online education experts, oncologists and consumers, with the aid of a videographer with expertise in creating brief online clinical education courses. The training features didactic material on the prevalence, severity, clinical features and outcomes of FCR, description and modelling of the CIFeR intervention, and evidence supporting its efficacy, captured in short videos of the study team, and videos of clinicians and patients modelling intervention delivery. Training is hosted on the Thinkific platform and will take approximately 15 min to complete. The training is being piloted using think-aloud techniques with 5–6 oncologists not participating in the main study and will be further refined in line with feedback if necessary. As an alternative to the online course, if participants request, the course will also be run as a facilitated one on one educational session with video-conferencing, hosted by a member of the research team.

### Intervention

The CIFeR intervention components are described in Table 1. Further information is provided in the CIFeR phaseI/II pilot paper. As noted above, CIFeR is delivered by an oncologist or surgeon, during a face-to-face consultation or via TeleHealth if preferred during the COVID-19 pandemic or in rural contexts.

**Table 1:** Description of CIFeR components

Component	Description
FCR Normalisation	Reassurance given to patients that FCR is a common and normal phenomenon after diagnosis and treatment of breast cancer.
Provision of concrete prognostic information	1) Identifying if patients would like to know information about their recurrence risk and providing this information.
Take-home education sheet on red-flag recurrence symptoms	Including information and tips on when patients need to worry and what symptoms to worry about, simple strategies to use at home to manage worry, and online resources for managing FCR.
Take home information sheet on strategies to manage worry	Distraction, meditation, mindfulness, and reassurance, alongside other additional online resources to manage FCR.
Referral to a psychologist	If FCR is high, the patient would like additional help or if the clinician judges that the patient may need additional help referral to a psychologist is recommended.

### Quantitative data Collection

Using Proctor’s [15] implementation outcomes and Shepherd’s [17] article which described a conceptual approach to defining and operationalising implementation outcomes, we defined measures of success for the CIFeR_2 study (see Table 2).

**Table 2:** Measuring CIFeR implementation success based on Proctor Outcomes

Outcome	Proctor et al. [15] Definition	Definition applied to CIFeR Study	Timing of measurement	Measurement Source
Adoption	“The intention, initial decision, or action to try or employ an innovation or evidence-based practice”	The delivery of CIFeR intervention to patients by participating clinician	T2, T3	3-month post-training questionnaire, interview
Acceptability	“The perception among implementation stakeholders that a given treatment, service, practice, or innovation is agreeable, palatable, or satisfactory”	The extent to which participating clinicians perceive CIFeR to be acceptable	T2 and T3	3- and 6-month post-training questionnaire, interview
Appropriateness	“The perceived fit, relevance, or compatibility of the innovation or evidence based practice for a given practice setting, provider, or consumer; and/or perceived fit of the innovation to address a particular issue or problem.”	The extent to which participating clinicians report CIFeR to fit their practice setting and their patients	T2 and T3	3- and 6-month post-training questionnaire, interview
Cost	“The cost impact of an implementation effort. This includes cost of the intervention itself, the implementation strategy used and costs based on location of service setting.”	The cost of CIFeR determined by time required to deliver CIFeR, proportion of patients referred to psychosocial health professionals and printing of CIFeR leaflets	T0, T1 and T2?	Baseline, 3-and 6-month post-training questionnaire Recording of number of printed leaflets by study staff
Feasibility	“The extent to which a new treatment, or an innovation, can be successfully used or carried out within a given agency or setting”	Proportion of participating clinicians who report CIFeR to be feasible or very feasible in their practice 3 and 6 months after the CIFeR training	T2 and T3	3 and 6-month post-training questionnaire
Fidelity	“The degree to which an intervention was implemented as it was prescribed in the original protocol or as it was intended by the program developer”	The degree to which participating clinicians deliver all 5-components of the CIFeR intervention.	T1	Checklist comprises 5 items assessing fidelity
Penetration	“The integration of a practice within a service setting and its subsystems.”	The extent to which informed clinicians agreed to participate in the study.	T1	Manual calculation
Sustainability	“The extent to which a newly implemented treatment is maintained or institutionalized within a service setting’s ongoing, stable operations.”	The extent to which clinicians have delivered CIFeR within the last 3-months of the intervention period.	T3	6-month post-training questionnaire, interview

#### Primary outcome

The primary outcome of the CIFeR_2 study is adoption (percentage of participating oncologists or surgeons who report offering the CIFeR intervention to at least one early stage/curable breast cancer patient attending a follow-up appointment 3 months after receiving the CIFeR training). The CIFeR intervention will be defined as adopted if ≥ 50% of oncologists or surgeons deliver the CIFeR intervention to at least one early stage/curable breast cancer patient in that timeframe, whereas the intervention will be deemed not adopted if < 50% of oncologists deliver CIFeR in that timeframe.

#### Secondary outcomes

Secondary outcome measures are.

1. Acceptability (percentage of participating oncologists or surgeons who report CIFeR to be acceptable or very acceptable) 3 and 6 months after the CIFeR training). This is measured on a 4-point Likert scale where 1 = not acceptable, 2 = moderately acceptable, 3 = acceptable and 4 = very acceptable.
2. Appropriateness (percentage of participating oncologists or surgeons who report CIFeR to be appropriate or very appropriate to their patients) 3 and 6 months after the CIFeR training). This is measured on a 4-point Likert scale where 1 = not appropriate, 2 = slightly appropriate, 3 = appropriate and 4 = very appropriate.
3. Feasibility (proportion of participating oncologists who report CIFeR to be feasible or very feasible in their practice 3 and 6 months after the CIFeR training). This is measured on a 4-point Likert scale where 1 = not feasible, 2 = slightly feasible, 3 = feasible and 4 = very feasible.
4. Fidelity (proportion of the first three patients receiving CIFeR to whom all 5 components of CIFeR are delivered, as assessed by oncologist-completed checklist self-reported 3 months after the CIFeR training). The checklist comprises 5 items assessing fidelity to each of 5 CIFeR intervention components, with yes/no response options and open-ended questions eliciting reasons for not delivering components if that occurs.
5. Penetration: (percentage of oncologists or surgeons informed about CIFeR who express interest in using CIFeR in their clinical practice and agree to participate in the implementation study). This will be recorded as the difference between the total number of oncologists or surgeons informed about the CIFeR implementation study (recorded by the study research assistant) and how many agree to participate.
6. Sustainability: (proportion of participating oncologists or surgeons who report having used CIFeR with at least one patient within the last 3 months, 6-months after the CIFeR training.
7. Costs (Oncologist or surgeon-estimated mean time taken to deliver CIFeR in minutes; costs of printing CIFeR leaflet (recorded by study staff); Oncologist or surgeon-estimated proportion of patients referred to psychologists or other psychosocial health professionals for help with FCR; CIFeR will be determined to be low cost if the time taken to deliver the intervention is < 10 min, printing costs are < $1 per leaflet, and proportion of patient referrals to psychosocial health professionals does not increase.
8. Barriers and facilitators to implementation generated from semi-structured qualitative interviews with oncologists or surgeons 6 months after the CIFeR training.
9. Self-efficacy: (change in oncologists’ or surgeons’ self-efficacy to manage FCR scores from baseline to post-training, 3 and 6 months after the CIFeR training). This will be measured by the 12-item Self-efficacy Questionnaire (SE-12) [18], adapted to target self-efficacy in managing FCR in patients, with 4-point response scales.

Data on demographics and professional characteristics will be collected at baseline. Participants will also be asked to report estimated proportion of patients referred to psychologists or other psychosocial health professionals for help with FCR, and the number of patients to whom they have delivered CIFeR at baseline and 3 and 6-month assessment points.

### Semi-structured interview

At 6 months post CIFeR training, participants will be contacted to arrange a semi-structured telephone interview at a time convenient to them, to elicit their feedback about barriers and facilitators to using CIFeR in routine clinical practice, conducted by a trained qualitative researcher. Open questions will elicit discussion about the CIFeR training, the CIFeR intervention as a whole, specific components of the intervention, barriers and facilitators to implementing CIFeR, and ideas to improve translation of CIFeR into routine practice (Supplementary File 5). Probing questions will be used to deepen and extend responses. The recorded interviews will be transcribed verbatim.

### Sample size

The sample size was determined using the power-based approach for the primary endpoint, assuming that the intervention may be adopted if H1: p > 50% (greater than 50% of consenting clinicians offer CIFeR to at least 1 patient at 3-months follow-up), and that H0: p < 30%, a level below which the intervention will not be regarded as adopted. If 30% is assumed for the participation rate under the null and 50% under the alternative, then based on a one-sided alpha = 5% and a power of 80%, the estimated sample size is n = 39. The intervention would be regarded as adopted, if at least 17 out of 39 clinicians offer CIFeR to at least 1 early stage/curable breast cancer patient by 3 months. Assuming a drop-out rate of 25% then 50 clinicians will need to be recruited to meet the primary endpoint of the study.

### Data handling

Study data will be recorded on the password protected Qualtrics server. All required data entry fields will be completed. All completed questionnaires, audio-recordings and transcripts will be managed centrally at the University of Sydney. Electronic data will be collected securely by the Qualtrics database, and only the chief investigator or site principal investigator will have access to the study data. All information will be stored securely for seven years as per NHMRC and will only be available to staff directly involved with the study.

Non-identified data will be analysed by the core research team (chief investigator, principle investigator and sub-investigators), with the possible assistance of additional research staff or research students. A Clinical Study Report will be issued which may form the basis of a manuscript intended for publication.

All data collected for, used in, or generated by this project will be disposed by secure methods after seven years from the completion of the study. Paper files will be shredded and computer files will be deleted. Any major changes to the protocol will be updated to participants and relevant ethics committee.

### Statistical analyses

Measures of Proctor outcomes (e.g. intervention adoption, acceptability) will be reported using descriptive statistics including proportions, means and standard deviations. Baseline demographics will be summarized in table format. Repeated measure t-tests will be used to examine changes in oncologist self-efficacy scores pre- and post-intervention. Proctor outcomes will be summarised using descriptive statistics. Exploratory predictors of higher adoption rates will be examined using linear models. Kolmogorov-Smirnov and Shapiro-Wilk tests of normality (tests statistic, degrees of freedom, p-value) will be performed.

As there are no pre-specified instructions available for handling missing data for the SE-12, the averages for the remaining items for the scale in question will be calculated with the adjusted denominator. Missing data from the questionnaires will be descriptively reported and all available data will be included for analysis.

Qualitative data will be analysed using Framework analysis [19]. Line-by-line coding will be conducted on three transcripts by the research team to develop the preliminary coding framework, which will be iteratively refined following review of subsequent transcripts. Over-arching themes and sub-themes will be developed to summarize the data. Differences in researcher interpretation of the data will be resolved through discussion. Themes arising from medical and radiation oncologists and surgeons will be compared. We will use the consolidated criteria for reporting qualitative research (COREQ) to guide reporting [20].

### Recruitment timeline

Recruitment has started as of March 2022 and recruitment is in process of finishing with final follow-up to be sent in May 2023.

## Discussion

### Theoretical significance

Given there are currently no clinician-delivered interventions to address FCR, the CIFeR_2 implementation study advances the field by representing and solidifying the evidence for the beneficial and appropriate use of a clinician-lead educational intervention for patient FCR within the context of follow-up clinics. Additionally, the current study will provide a guide of implementation efforts, as well as provide a greater understanding of the barriers and facilitators of implementing CIFeR in routine care. Implementation studies are increasingly being used to identify and address barriers early in implementation efforts, to ensure successful integration of evidence into practice [21].

### Clinical significance

Successful completion of the CIFeR_2 Study will provide proof-of-principal that doctors can address worries regarding FCR with their patients, and that CIFeR can be feasibly introduced into routine care. CIFeR_2 addresses psycho-oncology workforce shortages through increased training of oncologists and oncology surgeons to deliver care to patients with mild-moderate FCR. Providing a brief intervention that incorporates self-management has the potential to decrease health service utilisation by patients with untreated FCR. If this project is successful, we will have a user-tested online training module that can be delivered to oncologists and oncology surgeons across Australia. We will also have data on the use and perceived utility of this training module that can guide further refinement of the training. We will have a rich set of quantitative and qualitative data on the factors required for success in implementing a clinician-delivered intervention for FCR (CIFeR). These data will allow us to further refine the CIFeR intervention and the system, clinician and patient focused strategies that will optimise the likelihood that it will be effective and implemented in routine care in the long-term. Finally, the five key components of the intervention are tumour site agnostic and thus the CIFeR intervention could be readily adapted to other tumour streams where FCR is a common and problematic phenomenon (E.g., childhood haematological malignancies, adolescent sarcomas, testicular cancers and ovarian cancers). Future research is needed to explore if CIFeR can be as effectively used by diverse clinical and allied health specialists after adequate training.

## Conclusion

FCR continues to impact a large proportion of cancer survivors, and increasingly stepped care interventions are required to address this issue in patients depending on their level of worry. The CIFeR intervention is a brief and low-cost intervention that has been shown to be feasible, acceptable and potentially effective in preventing FCR, as well as reducing its severity and duration in patients who develop FCR. This research will explore whether oncologists and oncology surgeons more widely will take up CIFeR after formal education and training on how to manage FCR in routine clinical practice, providing valuable information about the practicalities of implementing this beneficial intervention into routine clinical care and telehealth.

## Electronic supplementary material

Below is the link to the electronic supplementary material.

Supplementary Material 1

Supplementary Material 2

Supplementary Material 3

Supplementary Material 4

Supplementary Material 5

## Data Availability

Not applicable. Data has not been presented in this paper as this is a study protocol paper.

## Abbreviations

FCR: Fear of cancer recurrence
CIFeR: The Clinician Intervention to Reduce Fear of Recurrence
SE-12: Self-efficacy Questionnaire
