# Supplementary material for: Protocol of an implementation study of a clinician intervention to reduce fear of recurrence in cancer survivors (CIFeR_2 implementation study)
Source: BMC Med Educ. 2023 May 5;23:312. doi: 10.1186/s12909-023-04279-0 (PMC10161179; doi:10.1186/s12909-023-04279-0)
Supplement: Supplementary file 1 — Supplementary Material 1 [file 12909_2023_4279_MOESM1_ESM.docx]

Open questions will be used initially and may include the following questions:

| **Topic** | **Initial open questions** | **Possible probing questions** |
| --- | --- | --- |
| Training | How useful did you find the CIFeR training? | Positives, negatives, recommended changes.  Comments on format, use of audio-visual material, ease of access, depth and breath of coverage |
| CIFER intervention as a whole | How have you found using the CIFeR intervention? | What things did or didn’t you like about the CIFeR intervention?  Did you use all aspects of the CIFeR intervention, or only some? Why?  How did the the CIFeR intervention affect your clinic workload and flow?  Would you recommend the CIFeR intervention to other clinicians? Why/Why not?  What changes would you recommend to the CIFeR intervention? |
| Specific components (if not already covered) | Most useful and most challenging parts of the CIFeR intervention?  What changes would you recommend for the intervention? | Normalisation  Prognostic discussion  Take-home education sheet  Strategies for managing worry  Psychology referral |
| Barriers and facilitators | What made it hard or easy to use the CIFeR intervention? | Training  Oncologist factors  Patient factors  System factors |
| Translation to practice | What would you recommend to ensure the CIFeR intervention was implemented effectively? | What would make you more or less likely to use this intervention in your routine clinical practice moving forward? |
