# Supplementary material for: Protocol of an implementation study of a clinician intervention to reduce fear of recurrence in cancer survivors (CIFeR_2 implementation study)
Source: BMC Med Educ. 2023 May 5;23:312. doi: 10.1186/s12909-023-04279-0 (PMC10161179; doi:10.1186/s12909-023-04279-0)
Supplement: Supplementary file 2 — Supplementary Material 2 [file 12909_2023_4279_MOESM2_ESM.docx]

# 3-month (post-training mid-point) and 6-month (post-training) follow-up questionnaire (identical at each time point)

[Insert 3 or 6] MONTH POST-TRAINING QUESTIONNAIRE Date: ___/___/___

1. I have used the CIFeR intervention for at least one Agree Disagree

patient during a routine follow up appointment

1. To how many patients did you deliver the CIFeR intervention **in the past 3 months**? _______ patients
2. How many patients did you refer to a psychosocial health service **in the last 3 months**

for help with FCR?  0% 1-25% 26-50% >50%

1. On average, how much time did it take you to deliver CIFeR in your consultations? _______minutes
2. Please indicate how much you agree with each of the statements below. Please mark [X] only one box in each line.

|  | Not  acceptable | Moderately  Acceptable | Acceptable | Very  acceptable |
| --- | --- | --- | --- | --- |
| I found CIFeR to be _____________ |  |  |  |  |
|  | Not  appropriate | Slightly  Appropriate | Appropriate | Very  appropriate |
| I found CIFeR to be _____________ |  |  |  |  |
|  | Unfeasible | Slightly  feasible | Feasible | Very  feasible |
| I found CIFeR to be an _________  addition to my follow up consultations |  |  |  |  |

6.Please indicate how confident you feel now in successfully addressing the following:

|  | Not confident | A little confident | Quite confident | Very confident |
| --- | --- | --- | --- | --- |
| Identifying FCR as a concern that the patient wishes to address/discuss during the conversation |  |  |  |  |
| Encouraging the patient to express her feelings or concerns surrounding FCR |  |  |  |  |
| Listening attentively to the patient talking about FCR without interrupting or changing the focus |  |  |  |  |
| Demonstrating empathy – verbally and non-verbally (e.g. supportive tone, eye-contact) during FCR discussions. |  |  |  |  |
| Structuring the conversation about FCR with the patient (e.g. checking understanding of prognosis, symptoms indicating recurrence) |  |  |  |  |
| Clarifying the patient’s level of knowledge and understanding to communicate right amount of information |  |  |  |  |
| Devising a plan to manage FCR based on shared decision making and using psychosocial supports (i.e. psychologists, breast care nurses) |  |  |  |  |
| Closing the conversation |  |  |  |  |

| 7. Please select all of the relevant strategies listed below, that you used to manage FCR in your patients in the past few months: | |
| --- | --- |
| Information (e.g. Likelihood of remaining disease free) |  |
| Medical Investigations (e.g. Scans and blood tests) |  |
| Stress management techniques |  |
| Referral to psychosocial support (e.g. psychologist and social worker) |  |
| Psychotropic medications |  |

Other (please specify) ____________________________

8. What proportion of CIFeR intervention were delivered via telehealth (either video-conference or telephone)?

>10%, 10-25% 26-50% >50%

9. Finally, using the checkboxes below, please indicate:

|  | Not at all | Somewhat | Moderately | Very |
| --- | --- | --- | --- | --- |
| How challenging you find helping patients dealing with FCR? |  |  |  |  |
| How interested you are in receiving training for managing FCR in patients? |  |  |  |  |

**Thank you for completing this survey**
