# Supplementary material for: Protocol of an implementation study of a clinician intervention to reduce fear of recurrence in cancer survivors (CIFeR_2 implementation study)
Source: BMC Med Educ. 2023 May 5;23:312. doi: 10.1186/s12909-023-04279-0 (PMC10161179; doi:10.1186/s12909-023-04279-0)
Supplement: Supplementary file 3 — Supplementary Material 3 [file 12909_2023_4279_MOESM3_ESM.pdf]

INTERVENTION CHECKLIST (first three patients)

IMMEDIATELY POST-INTERVENTION

We would like your feedback on which components of CIFeR you were able to deliver.

Please indicate using the checkbox **[X]** how many of the components below were covered in the discussion with your patient today.

Date: ___/___/___

Patient seen:

First CIFeR patient ☐ Second CIFeR patient ☐ Third CIFeR patient ☐

1) Normalisation of FCR – discussing with and reassuring the patient that FCR is normal and occurs frequently among survivors

   Yes ☐ No ☐

2) Prognostic information re cancer stage, type and unique risk of recurrence

   Yes ☐ No ☐

3) Information about follow-up and symptoms most likely to indicate recurrence

   Yes ☐ No ☐

4) Information sheet on suggested strategies to manage FCR to give to patient

   Yes ☐ No ☐

5) Referral to psychologist or other psychosocial health professional if FCR is severe and patient would like additional help

   Yes ☐ No ☐

Please indicate reasons why any components were not delivered today:

____

____

____

How was the intervention delivered?

Face-to-Face (in person) ☐ Video conference ☐ Telephone (audio only) ☐

We thank you for your time spent completing this form.
Please remember to **complete this form for each of the first three patients** you complete CIFeR with.
