# Supplementary material for: Protocol of an implementation study of a clinician intervention to reduce fear of recurrence in cancer survivors (CIFeR_2 implementation study)
Source: BMC Med Educ. 2023 May 5;23:312. doi: 10.1186/s12909-023-04279-0 (PMC10161179; doi:10.1186/s12909-023-04279-0)
Supplement: Supplementary file 4 — Supplementary Material 4 [file 12909_2023_4279_MOESM4_ESM.docx]

Immediately Post-Training Questionnaire

1. **Now that you have completed the training, please indicate how confident you feel in successfully addressing the following:**

|  | Not confident | A little confident | Quite  confident | Very confident |
| --- | --- | --- | --- | --- |
| Identifying FCR as a concern that the patient wishes to address/discuss during the conversation |  |  |  |  |
| Encouraging the patient to express her feelings or concerns surrounding FCR |  |  |  |  |
| Listening attentively to the patient talking about FCR without interrupting or changing the focus |  |  |  |  |
| Demonstrating empathy – verbally and non-verbally (e.g. supportive tone, eye-contact) during FCR discussions |  |  |  |  |
| Structuring the conversation about FCR with the patient (e.g. checking understanding of prognosis, symptoms indicating recurrence) |  |  |  |  |
| Clarifying the patient’s level of knowledge and understanding of FCR to communicate right amount of information |  |  |  |  |
| Devising a plan to manage FCR based on shared decision making and using psychosocial supports (i.e. psychologists, breast care nurses) |  |  |  |  |
| Closing the conversation about FCR |  |  |  |  |
| Estimating risk of recurrence |  |  |  |  |

1. **Please tell us how much you agree or disagree with the following statements:**

|  | Strongly disagree | Disagree | Undecided | Agree | Strongly agree |
| --- | --- | --- | --- | --- | --- |
| The training modules provided **information** that was practical and useful |  |  |  |  |  |
| The training modules taught me **skills** that was practical and useful |  |  |  |  |  |
| The training modules made me reflect on my current ways of communicating with patients about FCR management and referral |  |  |  |  |  |
| The format of the training modules was appropriate |  |  |  |  |  |
| The length of time of training modules was appropriate |  |  |  |  |  |

1. **How comfortable are you explaining prognostic information to patients?**

| Not comfortable | A little comfortable | Quite  comfortable | Very comfortable |
| --- | --- | --- | --- |
|  |  |  |  |

Do you have any suggestions on improving the FCR training (content, format or delivery)? Please elaborate:
