# Supplementary material for: Protocol of an implementation study of a clinician intervention to reduce fear of recurrence in cancer survivors (CIFeR_2 implementation study)
Source: BMC Med Educ. 2023 May 5;23:312. doi: 10.1186/s12909-023-04279-0 (PMC10161179; doi:10.1186/s12909-023-04279-0)
Supplement: Supplementary file 5 — Supplementary Material 5 [file 12909_2023_4279_MOESM5_ESM.docx]

# BASELINE QUESTIONNAIRE

1. **Best contact number**: _______________
2. **Email address**: _________________
3. **Please provide your work address so that we can mail you a package containing 1) checklists to complete, 2) a script to assist with delivering the CIFeR intervention and 3) pamphlets to provide to patients:_______**
4. **Age** (years) : _______________
5. **Gender**:  Male  Female  Other
6. **Professional background**: Medical oncologist

Radiation oncologist

Medical oncology trainee

Radiation oncology trainee

Other (please specify)_ ___

1. **Years of experience in Oncology**: _______________________
2. **Please indicate your practice settings**: Urban  Regional  Rural

(tick all that are relevant) Private  Public

1. **Practice size:**  Large ( > 100 patients)  Small (< 100 patients)
2. **On average, how many breast cancer patients in follow-up do you see per month:** _________________
3. **On average, how long do your standard breast follow-up appointments take?** ___________(mins)
4. **In what percentage of follow-up consultations do you discuss FCR**

|  | 0% | 1 – 25 % | 26 – 50% | > 50 % |
| --- | --- | --- | --- | --- |

|  |  |  |  |
| --- | --- | --- | --- |

1. **What proportion of breast cancer survivors have you referred to a psychosocial health professional in the last 3 months for help with FCR?**

|  | 0% | 1 – 25 % | 26 – 50% | > 50 % |
| --- | --- | --- | --- | --- |
|  |  |  |  |  |

1. **Using the checkboxes below, please indicate:**

|  | < 10% | 10 – 25 % | 26 – 50% | > 50 % |
| --- | --- | --- | --- | --- |
| 1. The proportion of cancer survivors you see who require professional help for FCR? |  |  |  |  |
| The time taken during follow-up appointments spent discussing FCR related concerns? | 0 mins | 1-5 mins | 6-10 mins | > 10 mins |
|  |  |  |  |  |

1. **Please indicate how confident you feel in successfully addressing the following:**

|  | Not confident | A little confident | Quite  confident | | Very confident |
| --- | --- | --- | --- | --- | --- |
| Identifying FCR as a concern that the patient wishes to address/discuss during the conversation |  |  |  | |  |
| Encouraging the patient to express her feelings or concerns surrounding FCR |  |  |  | |  |
| Listening attentively to the patient talking about FCR without interrupting or changing the focus |  |  |  | |  |
| Demonstrating empathy – verbally and non-verbally (e.g. supportive tone, eye-contact) during FCR discussions |  |  |  | |  |
| Structuring the conversation about FCR with the patient (e.g. checking understanding of prognosis, symptoms indicating recurrence) |  |  |  | |  |
| Clarifying the patient’s level of knowledge and understanding of FCR to communicate right amount of information |  |  |  | |  |
| Devising a plan to manage FCR based on shared decision making and using psychosocial supports (i.e. psychologists, breast care nurses) |  |  |  | |  |
| Closing the conversation about FCR |  |  |  | |  |
| 1. **Please tick all of the relevant strategies listed below, that you use to manage FCR in your patients:** | | | | | |
| Information (e.g. Likelihood of remaining disease free) | | | |  | |
| Medical Investigations (e.g. Scans and blood tests) | | | |  | |
| Stress management techniques | | | |  | |
| Referral to psychosocial support (e.g. psychologist and social  worker) | | | |  | |
| Psychotropic medications | | | |  | |

Other (please specify) ____________________________

1. **Finally, using the checkboxes below, please indicate:**

|  | Not at all | Somewhat | Moderately | Very |
| --- | --- | --- | --- | --- |
| How challenging you find helping patients dealing with FCR? |  |  |  |  |
| How interested you are in receiving training for managing FCR in patients? |  |  |  |  |

**Thank you for completing this survey!**
